# Diabetes mellitus and hearing loss

**DOI:** 10.1186/s10020-023-00737-z

**Published:** 2023-10-24

**Authors:** Yuxin Deng, Sen Chen, Jun Hu

**Affiliations:** 1 Department of Endocrinology, Liyuan Hospital, Tongji Medical College, Huazhong University of Science and Technology, Wuhan, 430022 Hubei China; 2 Department of Otorhinolaryngology, Union Hospital, Tongji Medical College, Huazhong University of Science and Technology, Wuhan, 430022 China

**Keywords:** Diabetes mellitus, Hearing loss, Inner ear, Cochlea

## Abstract

Diabetes mellitus (DM) is a major disease threatening human health and its incidence is increasing year on year. As a chronic complication of DM, hearing loss mostly occurs undetectably. However, the mechanism of this diabetes-related hearing loss (DRHL) remains unclear and there is no effective clinical treatment. Studies of animal or human pathology show that DM causes damage to the blood vessels, spiral ganglion neurons, afferent nerve fibers, the organ of Corti, and the stria vascularis of the inner ear. In recent years, more advances in pathological research have revealed the possible mechanism of DRHL. In addition, a large number of clinical studies suggest that the duration and severity of DM are closely related to the incidence and severity of DRHL. This review focuses on the relationship between DM and hearing loss. The clinical audiological characteristics of diabetic patients, risk factors for DRHL, typical pathology, and potential interventions of DRHL are summarized. This will help reveal the pathogenesis and intervention approaches for DRHL.

## Introduction

Diabetes mellitus (DM) is a group of metabolic diseases characterized by hyperglycemia resulting from defects in insulin secretion, insulin action, or both. It can cause dysfunction of multiple target organs, including the eyes, kidneys, and heart, leading to diabetic retinopathy, diabetic nephropathy, and diabetic cardiomyopathy (Kollias and Ulbig 2010; Kanwar et al. 2011; Dillmann 2019). The inner ear is also one of the affected organs, with patients presenting with varying degrees of hearing loss. However, due to its insidious onset, patients may not be aware of this diabetes-related complication until their deafness becomes severe. Unfortunately, there is currently no effective clinical treatment, which places a huge psychological burden on diabetic patients and adversely affects their quality of life.

Hearing loss caused by DM can be referred to as diabetes-related hearing loss (DRHL), a term first proposed by Axelsson et al. (1978). This type of deafness is distinct from conductive deafness, which affects the external auditory canal or middle ear. It is classified as sensorineural hearing loss (SNHL) and primarily affects the nerve fibers or auditory sensory cells of the inner ear. In the process of exploring DRHL, pure tone audiometry (PTA), otoacoustic emissions (OAE), and auditory brainstem-evoked response (ABR) are often used to evaluate patients’ auditory function. Studies have shown that both type 1 diabetes mellitus (T1DM) and type 2 diabetes mellitus (T2DM) patients have worse hearing than normal people. OAE, which reflect the condition of hair cells of the inner ear, are significantly lower in patients with DM. The latency of the ABR, which reflects the electrical activity of the auditory nerve and its brainstem connections, is also prolonged in patients with DM (Teng et al. 2017; Akinpelu et al. 2014).

Due to differences between subjects and research methods, there are still contradictions between the characteristics of DRHL reported in different studies. Accordingly, the conclusions of these studies have not been consistent. Moreover, due to the inability to perform a biopsy in the human inner ear, the specific pathogenesis of DRHL remains unknown. Early pathological studies were mainly based on animal studies or autopsies, and the pathological manifestations mainly included degeneration of spiral ganglion neurons (SGNs), atrophy of the stria vascularis (SV), and degeneration of auditory hair cells. Recently, new advances have been made in the pathological study of DRHL (Lee et al. 2020; Seo et al. 2022). Therefore, in this review, we will discuss the relationship between DM and SNHL, summarize the various risk factors and protective factors of DRHL, then analyze and compare the pathological features of DRHL. We hope this review can draw attention to new ideas for the diagnosis and treatment of this auditory defect.

## The effect of DM on hearing

### DM increases the prevalence of hearing loss

Numerous studies have shown that DM increases the prevalence of SNHL in adults. In a large cross-sectional study (n = 37,773), Oh et al. showed that the prevalence of hearing loss was 17.3% and 6.5% in diabetic and non-diabetic patients, respectively (*P* < 0.05). When further stratified by age, it was found that the prevalence of deafness was also significantly higher in subjects with DM than in those without DM in almost all age groups. For example, the prevalence of mild deafness in the diabetic and non-diabetic groups was 4.95% and 1.28%, respectively (30–39 years age-group), 5.87% and 3.21% (40–49 years age-group), and 12.69% and 9.21% (50–59 years age-group) (Oh et al. 2014). In another small sample study (n = 130), Mozaffari et al. found that the prevalence of deafness in both T1DM patients (44.4%) and T2DM patients (45.1%) was approximately twice as high as in non-diabetics (20.0%) (Mozaffari et al. 2010). On this basis, several researchers conducted meta-analyses to explore the effects of T1DM and T2DM on hearing function. Teng et al. showed that the pooled odds ratio (OR) of the prevalence of deafness in patients with T1DM versus non-diabetics was 49.08 (95%CI 12.03–200.31) (Teng et al. 2017), while Mujica-Mota et al. calculated an OR of 7.73 (95%CI 3.32–17.98) (T1DM patients) (Mujica-Mota et al. 2018). Compared with Teng et al., the results reported by Mujica-Mota et al. may be more realistic because of the larger sample size this study included. In a study on T2DM, Horikawa et al. and Akinpelu et al. (2014, 2013).

This characteristic is also observed in the influence of DM on a specific type of hearing loss, sudden sensorineural hearing loss (SSNHL). This kind of hearing loss refers to a loss of 30 dB or more in three contiguous frequencies over less than three days. In a large sample cohort study (n = 53,112), Lin et al. (2012). However, this study did not distinguish the type of DM.

### DM aggravates hearing loss

DM can also aggravate hearing loss in adults. Mitchell et al. define “aggravate” as the progression of hearing loss greater than 5 dB over 5 years. The results showed that patients with T2DM had significantly higher rates of deafness progression than normal people, especially in newly-diagnosed diabetic patients, with an OR = 2.71 (95%CI 1.07–6.86) (Mitchell et al. 2009). Oh et al. (2014).

Similarly, DM increases the degree of SSNHL in adults. Shen et al. focused on the influence of glycosylated hemoglobin (HbA1c) level on SSNHL, and grouped deafness according to the mean hearing threshold. A mean hearing threshold greater than 80 dB HL was defined as total SSNHL. Their results showed that diabetic patients with SSNHL had a higher rate of total SSNHL, while the mean hearing threshold of patients without DM was mostly less than 80 dB HL (Shen et al. 2021).

### Clinical audiological characteristics of DRHL

At the same age, hearing sensitivity at specific frequencies was found to be worse in adults with DM than in normal people (Mitchell et al. 2009; Ma et al. 1998). Based on this finding, we further speculated that clinical characteristics of DRHL vary with age, that is, the main frequency affected will gradually progress from high frequency to low frequency as patients age.

For a relatively young population, the difference in deafness between diabetics and non-diabetics is more pronounced at high frequencies. In a small sample study (n = 60), Kurien et al. (1989). A similar phenomenon was observed by Das et al. in another cross-sectional study involving subjects aged 21 to 64 years. They found that the mean hearing threshold gap between the two groups was greatest at extra-high frequency (12 kHz: 71.11 ± 2.96 dB vs. 51.28 ± 3.01 dB) (Das et al. 2018).

For a relatively older population, the difference in hearing between diabetic and nondiabetic populations was more pronounced at relatively lower frequencies. Mitchell et al. measured the hearing level of those aged ≥ 49 years old. The results showed that the maximum difference was apparent at 2 kHz (29.9 ± 1.3 dB vs. 24.6 ± 0.5 dB, *P* = 0.0001) (Mitchell et al. 2009). Frisina et al. assessed hearing in subjects aged 59 to 92 years. They found that the differences measured at 1–4 kHz (mean gap = 12.3 dB) was significantly greater than that measured at 10–14 kHz (mean gap = 7.9 dB) (Frisina et al. 2006). Slightly different are the bone conduction threshold results measured by Chee et al. in participants aged 60 to 70 years. These results showed that the largest difference between the two groups was 5.4 dB (at 4 kHz) (*P* < 0.001) (Chee et al. 2022). This may be because Frisina et al. measured the mean of three frequencies, while Chee et al. measured the result of each frequency more precisely. Alternatively, it could be that the population studied by Chee et al. was a community-living group, which may have had relatively mild disease.

A few studies have reported the effects of DM on hearing in childhood and adolescence. However, the results are controversial. Some researchers believe that DM has no negative effects on hearing during this period (Sieger et al. 1983), while others believe that the effects of DM on hearing during this period are similar in some respects to the effects in adulthood. For example, Okhovat et al. (2011). However, due to the small sample sizes of these studies, it is difficult to draw definite conclusions regarding any correlations between DM and hearing loss in this age-group. Therefore, we mainly discuss the impact of DM on hearing in adults.

## Risk factors for DRHL

Studies have shown that DRHL is influenced by a variety of risk factors, such as the duration and severity of DM, and the age of the DM patient population.

### Duration of DM and DRHL

#### Duration of DM and incidence of DRHL

Duration of DM is an important variable affecting the incidence of DRHL and is positively correlated with the incidence of hearing loss (Mitchell et al. 2009; Hosseini et al. 2020; Gupta et al. 2019). According to statistics, patients with T1DM have twice the risk of DRHL when the DM duration is 4–12 years compared to non-diabetic individuals (Mujica-Mota et al. 2018). For patients with T2DM, researchers conducted a more detailed stratified analysis. The study of Al-Rubeaan et al. (which included subjects aged 30–60 years) showed that the incidence of DRHL in patients with a DM duration of 5 years or less was 47.1%, while the incidence of DRHL in patients with DM duration of 6–10 years was 53.6%. When the course of DM exceeded 10 years, this percentage increased to 62.6% (Al-Rubeaan et al. 2021). Another study by Srinivas et al. showed that the incidence of DRHL in subjects with DM duration of 6–10 years was almost twice that in subjects with DM duration of 5 years or less (68.5% vs. 35.71%) (Srinivas et al. 2016). This result may be related to the small sample size of their study.

#### Duration of DM and severity of hearing loss

Whether a longer duration of DM exacerbates hearing loss is controversial. According to WHO 1980 grades of hearing impairment, hearing loss can be classified as mild (26 dB HL to 40 dB HL), moderate (41 dB HL to 55 dB HL), moderately severe (56 dB HL to 70 dB HL), severe (71 dB HL to 90 dB HL) or profound (> 91 dB HL). The results of a study by Mishra et al. showed that when the course of DM ranged from 1 to 3 years, only mild hearing loss occurred. When the duration of DM was 4–12 years, severe hearing loss began to occur among patients. And when the course exceeded 12 years, some patients developed severe and profound hearing loss (Mishra and Poorey 2019). Celik et al. and Das et al. (2018, 1996). Although Kurien et al. (1989). Other researchers did not observe a relationship between the duration of DM and the severity of deafness. Al-Rubeaan et al. (2021). This may be because people with T2DM are treated with hypoglycemic drugs to control their blood sugar, which delays the progression of hearing impairments. In addition, the sample sizes of these studies were too small, and further exploration is needed to draw firm conclusions.

### Severity of DM and DRHL

A correlation between the severity of DM and the level of hearing loss has also been reported. Blood sugar levels and the appearance of DM complications are most commonly used to represent the severity of DM.

#### DM complications and DRHL

As DM progresses, many complications occur. One study found that diabetic patients with complications had worse hearing thresholds than those without complications (29.5 ± 12 dB vs. 19.7 ± 10 dB, *P* < 0.05) (Kurien et al. 1989). Among all comorbidities, the hazard ratio (HR) of retinopathy was found to be the highest, at 1.967 (95%CI, 1.141–3.389) (Lin et al. 2012). This may suggest that there is a high correlation between the pathological changes of the retina and the inner ear. However, no studies have explored the pathological relationship between these two organs, and most studies are still in the clinical stage without biopsy. Alizadeh et al. found that the degree of diabetic retinopathy was positively associated with the prevalence of hearing loss. The incidence of hearing loss at high frequency was 1.17 times higher in the severe retinopathy group (71.4%) than in the mild-to-moderate retinopathy group (61.0%). At the frequency of speech, the ratio of the incidence of DRHL in the two groups was close to three times (31.4% vs. 11.4%) (Alizadeh et al. 2022). In addition, Ooley et al. (2017).

#### Glycosylated hemoglobin levels and DRHL

Glycosylated hemoglobin (HbA1c) is clinically recognized as a better reflection of plasma glycemic status over the preceding 2 to 3 months and is a reliable diagnostic biomarker for DM.

HbA1c levels were found to be positively correlated with the incidence of hearing loss in both T1DM and T2DM patients. In a study on T1DM, Schade et al. (2018). In another study on T2DM, Al-Rubeaan et al. showed that patients with poor glycemic control (HbA1c ≥ 8%) had a higher rate of hearing loss than those with good glycemic control (HbA1c < 8%) (62.9% vs. 48.3%) (Al-Rubeaan et al. 2021). Srinivas et al. (2016). However, Cruickshanks et al. (2015).

Other researchers have found that the HbA1c level was associated with SNHL in the non-diabetic population (Ooley et al. 2017). Kang et al. observed that, compared to subjects with low HbA1c levels (5.3% ± 0.2%), subjects with high HbA1c levels (6.1% ± 0.2%) had a 1.253-fold increased risk of hearing loss and a 3.4–6.4 dB increase in hearing threshold (Kang et al. 2016). Zeng et al. (2022).

#### Fasting blood glucose and DRHL

The relationship between fasting blood glucose (FBG) and DRHL remains unclear. Some researchers believe that there is an association between high FBG levels and the development of SNHL (Das et al. 2018; Mishra and Poorey 2019). In a small sample study (n = 50), Srinivas et al. found that SNHL was present in about 79% of T2DM patients with FBG > 7.0 mmol/L, and in 28% of T2DM patients with FBG < 7.0 mmol/L (*P* = 0.0283) (Srinivas et al. 2016). However, results from Mozaffari et al. (2010). One potential explanation is that hearing loss is a gradual process in most cases, and FBG levels that reflect a single point in time do not necessarily correlate with hearing function.

Other researchers used the treatment as a proxy for DM severity. Their findings showed that compared with diabetic patients treated with single antidiabetic drugs, patients treated with triple antidiabetic drugs had a significantly increased risk of developing SSNHL, with an HR of 2.060 (95%CI, 1.051–4.037) (Lin et al. 2012). However, the association between oral hypoglycemic or insulin treatment and the incidence of hearing loss is unclear at present (Shafiepour et al. 2022).

### Other factors and DRHL

#### Age and DRHL

A large number of studies have shown that hearing thresholds and the prevalence of hearing loss increase with age in DM patients (Mitchell et al. 2009; Kurien et al. 1989). In a study involving patients with DM aged 31–65 years, Srinivas et al. found that the prevalence of SNHL was lowest in the 31–35 age group (0%) and highest in the 46–65 age group (78.12%) (Srinivas et al. 2016). On this basis, Chee et al. and Uchida et al. (2010). We speculated that when the age is over 65 years old, the presbycusis factor plays a more significant role in the auditory system.

Lin et al. drew a similar conclusion in the study of SSNHL. They found that compared with a non-diabetic group, the incidence of SSNHL in the DM group was highest in the 50–64 age group (1.67 per 1,000 person-years vs. 0.90 per 1,000 person-years, *P* = 0.0009), followed by the 35–49 age group (1.04 per 1,000 person-years vs. 0.47 per 1,000 person-years, *P* = 0.0037). However, there was no significant difference in the incidence of SSNHL between the diabetic and non-diabetic populations in the age groups under 35 years and over 65 years (Lin et al. 2012). Therefore, these findings can distinguish DRHL from presbycusis, which occurs in people over the age of 60 and progressively degrades the auditory system with age. For people over the age of 65, their hearing loss may be influenced by multiple factors, including genetics, aging, noise, and smoking. At this stage, blood sugar no longer plays a decisive role. In contrast, hearing loss is rare in normal people under 60, where hyperglycemia is enough to make a difference in hearing thresholds between the two groups.

#### Gender and DRHL

Whether the relationship between DM and deafness differs by gender is currently unknown. Some groups believe that female diabetic patients have poorer hearing and a higher risk of hearing loss than males (Taylor and Irwin 1978). A large cohort study (n = 16,140) showed that the incidence of hearing loss was 29.64 per 1,000 person-years in women with DM and 25.23 per 1,000 person-years in men with DM (Wang et al. 2022). Conversely, other groups have reported that male diabetic patients are more susceptible to hearing impairment (Al-Rubeaan et al. 2021; Ren et al. 2017; Bainbridge et al. 2011). This may be related to the prolonged exposure of many male subjects to noisy conditions associated with industrial work or entertainment. However, most studies have not found a link between gender and DRHL (Lin et al. 2012; Schade et al. 2018).

#### Noise and DRHL

Long-term noise exposure is harmful to hearing in normal people; however, the effects of noise on DRHL have only been reported in a few animal studies. The findings of McQueen on T2DM rats suggested that the basilar membrane thickness of stria vascularis capillaries in both lean and fat diabetic rats after noise exposure were thicker than that of diabetic rats without noise interference (diabetic lean noise group = 0.1479 μm; control obese quiet group = 0.0930 μm; diabetic obese noise group = 0.2071 μm; control lean quiet group = 0.0930 μm) (*P* < 0.05) (McQueen et al. 1999). Han et al. and Raynor et al. also discussed differences in the effects of noise on hearing in diabetic and non-diabetic mice. Their results showed that compared with non-diabetic mice, T1DM mice and T2DM mice had greater hearing threshold shifts (the difference between the values before and after noise exposure) and more hair cell loss (*P* < 0.05) (Han et al. 2018; Raynor et al. 1995). These findings suggest that the auditory system of diabetic animal models is more vulnerable to noise trauma than that of non-diabetic animal models. Therefore, people with DM need to take more care to protect their hearing from noise exposure.

#### Vitamins and DRHL

Many researchers have studied the effects of vitamins on DM or hearing (Hatano et al. 2008; Curhan et al. 2015; Akyay et al. 2021; Schmitz et al. 2012). However, for effects of DRHL, only vitamin D has been reported. Vitamin D is a lipid-soluble vitamin that plays an important part in regulating bone homeostasis. Since Norman et al. (1980), studies on the relationship between vitamin D levels and diabetic complications gradually emerged (Jung et al. 2016; Luo et al. 2017). Hosseini et al. found that among people with T2DM, those with low vitamin D levels (less than 30 mg/dL) had 2.25 times the risk of hearing loss compared to those with normal vitamin D levels (Hosseini et al. 2020). Bener et al. also found that T2DM patients with impaired hearing had significantly lower vitamin D levels than T2DM patients with normal hearing (19.40 ± 0.71 ng/mL vs. 22.65 ± 9.280 ng/mL) (Bener et al. 2018). At present, findings regarding the mechanism of the effect of vitamin D on hearing are not consistent. More studies are needed to explore the protective mechanisms of vitamin D against DRHL and the effects of other vitamins on DRHL.

#### Proton pump inhibitors and DRHL

Proton pump inhibitors (PPIs) are the most effective acid-suppressing agents used to treat and prevent various gastrointestinal diseases. However, recent studies have found that PPI use can cause adverse effects on the nervous system, including hearing loss (Makunts et al. 2019; Kekilli et al. 2014). In a large case-control study (n = 6,891), Yee et al. (2022). This suggests that DM patients should be more careful when choosing PPI drugs.

#### Loop diuretics and DRHL

Loop diuretics, also known as high-efficacy diuretics, are a class of medicine that promotes discharge of electrolytes and water from the body, increases urine output, and eliminates edema. Schwartz et al. were the first to discover that these agents have ototoxic side effects (Schwartz 1970; Lloyd-Mostyn and Lord 1971). The hearing loss they cause is usually temporary (Venkateswaran 1971), but for some patients it is permanent (Quick and Hoppe 1975). However, the mechanism of hearing loss caused by this class of drugs is unclear. In addition, loop diuretics have also been reported to increase blood glucose levels and reduce the utilization of glucose by external adipose tissue (Weller and Borondy 1967). However, the side effect may be reduced after the patient discontinues use of the agent (Breckenridge et al. 1967). To date, there have been no studies on loop diuretics and DRHL. There is no evidence that long-term use of loop diuretics can induce DRHL by raising blood glucose.

## Typical pathological changes of DRHL

**Fig. 1:**
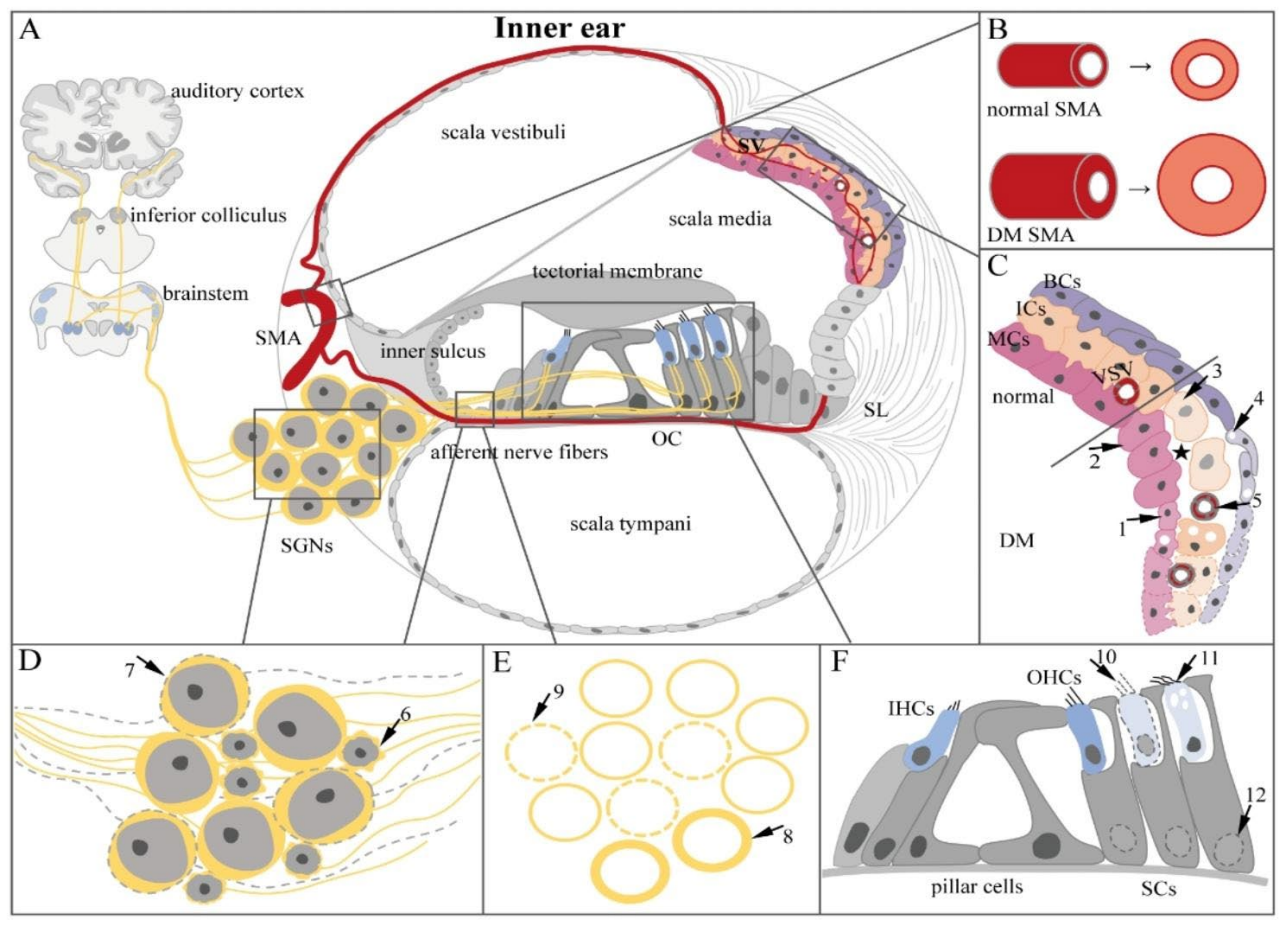
(**A**) The physiological structure of the inner ear up to the auditory central system. (**B**) Typical pathological changes of the SMA damaged by DM. (**C**) Typical pathological changes of the SV and VSV damaged by DM. (**D**) Typical pathological changes of the spiral ganglion damaged by DM. (**E**) A cross-section showing typical pathological changes of cochlear afferent nerve fibers damaged by DM. (**F**) Typical pathological changes of the OC damaged by DM DM: diabetes mellitus; SMA: spiral modiolar artery; SV: stria vascularis; VSV: vessels of the stria vascularis; MCs: marginal cells; ICs: intermediate cells; BCs: basal cells; SL: spiral ligament; SGNs: spiral ganglion neurons; OC: organ of Corti; OHCs: outer hair cells; IHCs: inner hair cells; SCs: supporting cells

The pathological study of DRHL is also constantly updated, and we summarize here the typical pathological changes of the inner ear in human temporal bones and animal models (Fig. 1; Table 1, and Table 2).

**Table 1:** Typical pathological changes of the inner ear in the DM group compared with the non-diabetic group: human temporal bone

Inner ear	Characteristic	Years	Author	Article
Cochlear vascular system				
SMA	Thickness of the vessel walls ↑	2010	Kariya et al.	(61)
SV	Significant atrophy	2005	Fukushima et al.	(62)
		2006	Fukushima et al.	(63)
	Mild atrophy was seen in two cases, normal in two cases	1971	Makishima et al.	(64)
	Total area ↓	2005	Fukushima et al.	(62)
VSV	Thickness of the vessel walls ↑	2005	Fukushima et al.	(62)
		2006	Fukushima et al.	(63)
		1961	Jorgensen et al.	(65)
	PAS-positive deposits	1961	Jorgensen et al.	(65)
		1971	Makishima et al.	(64)
**The organ of Corti**				
OHCs	Number ↓	2005	Fukushima et al.	(62)
		2006	Fukushima et al.	(63)
	Normal in three cases, slight degeneration in one case	1971	Makishima et al.	(64)
IHCs	Number NS	2005	Fukushima et al.	(62)
		2006	Fukushima et al.	(63)
**Cochlear nerve system**				
Spiral ganglion	Significant atrophy	1971	Makishima et al.	(64)
	The number of SGNs ↓	1971	Makishima et al.	(64)
	The number of SGNs NS	2005	Fukushima et al.	(62)
		2006	Fukushima et al.	(63)

**Table 2:** Typical pathological changes of the inner ear in the DM group compared with the non-diabetic group: animal models

Inner ear	Characteristic	Years	Author	Article
Cochlear vascular system				
SMA	Thickening of vessel wall ↑;	2022	Seo et al.	(8)
	lumen diameter ↑.			
SV	Protrusion or condensation of marginal cells; swelling of intermediate cells; widening of the intercellular spaces.	1986	Tachibana et al.	(66)
		1986	Nakae et al.	(67)
	Thickness of the SV decreased; density of intermediate cells decreased.	2020	Lee et al.	(7)
VSV	Basement membrane thickness ↑	1999	McQueen et al.	(41)
**The organ of Corti**				
OHCs	Number NS	2020	Lee et al.	(7)
		1978	Gladney et al.	(70)
		1995	Raynor et al.	(43)
	Number ↓	1991	Triana et al.	(68)
		1992	Rust et al.	(69)
		1986	Nakae et al.	(67)
	Degeneration	2008	Lee et al.	(71)
		1986	Nakae et al.	(67)
IHCs	Number NS	2020	Lee et al.	(7)
	Degeneration	1986	Nakae et al.	(67)
**Cochlear nerve system**				
SGNs	Number ↓	2008	Lee et al.	(71)
		2020	Kang et al.	(73)
		2020	Lee et al.	(7)
Cochlear afferent nerve fibers	Number ↓;	2020	Kang et al.	(73)
	fiber endings are swollen and dislocated.			

**Abbreviations**: DM: diabetes mellitus; SMA: spiral modiolar artery; SV: stria vascularis; VSV: vessels of the stria vascularis; OHCs: outer hair cells; IHCs: inner hair cells; SGNs: spiral ganglion neurons; ↑: increase; ↓: decrease; NS: no significant difference

### Cochlear vascular system

#### Spiral modiolar artery

The spiral modiolar artery (SMA), a branch of the anterior inferior cerebellar artery, is the primary source of blood supply to the cochlea.

##### Human temporal bones

The SMA of humans affected by DM may present with thickening of the vessel wall. Kariya et al. measured the vessel wall thickness of the SMA in both diabetic and non-diabetic patients. Their results showed that the vessel wall thickness of diabetic patients was significantly greater than those of non-diabetic people (*P* < 0.001). Compared with T1DM patients, T2DM patients receiving insulin therapy exhibited thicker vessel walls and a higher vessel wall ratio (11.75 ± 3.26% vs. 8.38 ± 1.81%, *P* < 0.05) (Kariya et al. 2010).

##### Animal models

In animal model studies, Seo et al. specifically observed the SMA in mice with T2DM. They found that the mean vessel wall thickness was thicker in the diabetic group compared to the non-diabetic group (3.418 ± 0.328 μm vs. 2.388 ± 0.411 μm, *P* = 0.0143) (Seo et al. 2022).

#### Stria vascularis and the vessels of the stria vascularis

The stria vascularis (SV) is another vulnerable area for hyperglycemia. It is located in the outer wall of the cochlear duct and is composed of three types of cells: marginal cells (MCs), intermediate cells (ICs), and basal cells (BCs). They are surrounded by a dense plexus of intraepithelial capillaries, also known as vessels of the stria vascularis (VSV). They play a key role in maintaining endocochlear potential, ion transport, and endolymphatic balance.

##### Human temporal bones

Effects on the SV of humans affected by DM mainly manifest as atrophy. Fukushima et al. studied the temporal bones of diabetic and non-diabetic patients. The results showed that the degree of SV atrophy in diabetic patients was greater than that in nondiabetic patients (*P* < 0.0001). Among them, in patients with T1DM, the mean area of the SV in the apical turn was found to be half that of non-diabetic patients (Fukushima et al. 2005). And in patients with T2DM, the area of SV atrophy in patients treated with insulin was most obvious in the lower basal, lower middle, upper middle, and apical turns, while the area of SV atrophy in patients treated with oral hypoglycemic drugs was most obvious in the lower middle turn (Fukushima et al. 2006). However, Makishima et al. observed that only some DM patients developed mild atrophy of the SV (Makishima and Tanaka 1971).

The VSV of humans affected by DM may show significant thickening and even complete occlusion or disappearance of the lumen. In the study of Fukushima et al., they measured the mean thickness of walls of VSV in all subjects. The results showed that compared with non-diabetics, patients with T1DM and patients with T2DM had significant thickening of walls of VSV in all turns (*P* < 0.05) (Fukushima et al. 2005, 2006). In addition, Makishima et al. and Jorgensen et al. also showed that appreciable Periodic Acid-Schiff stain (PAS)-positive deposits were visible on the walls of VSV in diabetic patients (Makishima and Tanaka 1971; Jorgensen 1961).

##### Animal models

In diabetic animal models, researchers observed more pathological changes in the SV. Tachibana and Nakae used KK mice (a Japanese inbred mouse strain that can spontaneously develop DM) and non-obese diabetic (NOD) mice as animal models of T2DM and T1DM, respectively. At three months of age, the MCs of KK mice protruded into the endolymphatic space. With aging, there was marked swelling of the ICs and widening of the intercellular spaces. By the age of 8 months, MCs had atrophied and were replaced by swollen ICs and intercellular spaces. However, in age-matched nondiabetic animals, no gross pathological changes were observed in the SV (Tachibana and Nakae 1986). The SV of NOD mice also presented similar changes, which manifested as swelling of ICs, widening of the intercellular space, and degenerative changes of MCs (MCs protruding into endolymphatic space, and cytoplasm vacuolization or condensation) (Nakae and Tachibana 1986). Lee et al. (2020).

There are few studies on VSV in animal models of DM. In a study of mice with T2DM, McQueen et al. found that the basement membrane thickness of VSV of the diabetic group was increased compared with the non-diabetic group. Especially when both groups were exposed to noise, the difference in basement membrane thickness between the two groups was more statistically significant (0.2071 μm vs. 0.1215 μm, *P* < 0.01) (McQueen et al. 1999).

### Organ of Corti

The organ of Corti (OC) rests on the basilar membrane of the scala media and is composed of hair cells and supporting cells (SCs). Hair cells are the sensor of the hearing organ, which can convert mechanical energy into electrochemical potential signals. They are divided into one row of inner hair cells (IHCs) and three rows of outer hair cells (OHCs). Each hair cell contains about 100 stereocilia. When external sound waves cause the basement membrane of the cochlea to vibrate, hair cells and cilia move relative to the tectorial membrane. This movement generates electrical signals that are sensed by the hair cells and transmitted to axons and eventually to the brain. Mammalian hair cells are non-regenerative and damage to the hair cells can cause permanent hearing loss.

#### Human temporal bones

In most human studies, the effect of DM on the OC was mainly manifested by a further reduction in the number of OHCs, but no significant change in the IHCs. In the study of Fukushima et al., they observed that compared with non-diabetic patients, patients with T1DM had significantly greater loss of OHCs in the lower basal turn (*P* < 0.01). Meanwhile in patients with T2DM, loss of OHCs was observed in both the lower and upper basal turns (Fukushima et al. 2005, 2006). Makishima et al. observed the morphological structure of the OC, and they found that the morphology and structure of the OC in most patients were normal, and only one patient had slight degeneration of the hair cells (Makishima and Tanaka 1971).

#### Animal models

The OC in animal models affected by DM is slightly different from that in humans. In terms of the number of OHCs, some researchers observed that compared with non-diabetic mice, the number of OHCs in T1DM and T2DM mouse models was reduced (Nakae and Tachibana 1986; Triana et al. 1991; Rust et al. 1992), while others did not observe such significant changes (Lee et al. 2020; Raynor et al. 1995; Gladney 1978). In terms of the morphology of the OC, the basal turn of the cochlea in both T1DM mice and T2DM mice showed obvious degeneration, such as vacuolation of the IHCs and OHCs, and replacement of the OHCs by SCs (Nakae and Tachibana 1986), while the OC in non-diabetic mice showed no significant abnormality (Lee et al. 2008).

### Cochlear nerve system

#### Afferent nerve fibers of the cochlea

In recent years, DRHL has been suggested to be related to the state of the nervous system of the cochlea, especially afferent nerve fiber damage. However, relevant studies have only been reported in animal models (Lee et al. 2008). Cochlear afferent nerve fibers are fibers that transmit auditory signals from hair cells to the brainstem, and have their somas in the cochlear spiral ganglion. Studies have found that calretinin, a Ca^2+^ buffer protein, is non-uniformly distributed in cochlear afferent nerve fibers and hair cells. Cochlear afferent fibers with low calretinin content tended to contact the modiolar side of the IHC membrane, whereas those with high calretinin content tended to contact all sides of the IHCs (Sharma et al. 2018).

##### Animal models

In a study on mice with T1DM, Kang et al. (2020).

#### Spiral ganglion neurons

Spiral ganglion neurons (SGNs) are the primary afferent neurons of the auditory system, which transmit sound information from hair cells to the cochlear nucleus of the brainstem to produce hearing. Neurons can be divided into two types, type-I and type-II neurons. Type-I neurons account for 95% of the total. Each of these bipolar and myelinated fibers innervates only one IHC. Afferent Type-II neurons account for only 5% of the total number of spiral ganglions and innervate the OHCs of the cochlea (Fig. 1D).

##### Human temporal bones

The effect of DM on spiral ganglia in humans is still controversial. Makishima et al. observed that spiral ganglia in diabetic patients were significantly atrophied, and the number of SGNs was reduced by more than 50% (Makishima and Tanaka 1971). Fukushima et al. (2005, 2006). This may be because Makishima et al. reported results for each diabetic patient, whereas Fukushima et al. compared the difference in average results in the diabetic group versus the non-diabetic group.

##### Animal models

In research using animal models, the effect of DM on SGNs appears to be uncontroversial. Several researchers observed SGNs of T1DM mice or T2DM mice. In both groups they found that the number of SGNs in the diabetic mice was significantly reduced compared with the non-diabetic mice (Lee et al. 2008, 2020; Kang et al. 2020). In addition, Lee et al. (2020).

### Summary of pathological features

Numerous studies have shown that the damage caused by DM to the auditory organ is mainly concentrated in the inner ear. Therefore, we present a cartoon depicting the physiological structure of the inner ear (Fig. 1A) and the typical pathological features of DRHL, including the vascular system (Fig. 1B, C), the nervous system (Fig. 1D, E), and the organ of Corti (Fig. 1F). In the vascular system, the SMA damaged by hyperglycemia shows vessel wall thickening and increased lumen diameter (Fig. 1B) (Seo et al. 2022; Kariya et al. 2010). The typical pathological manifestations of the SV include reduced total area of the SV (dotted line) (Fukushima et al. 2005), atrophy (arrow 1) (Fukushima et al. 2005, 2006; Makishima and Tanaka 1971) or protrusion (arrow 2) of the cytoplasm of MCs (Tachibana and Nakae 1986; Nakae and Tachibana 1986), swelling of ICs (arrow 3) (Tachibana and Nakae 1986; Nakae and Tachibana 1986), widening of the intercellular spaces (★) (Tachibana and Nakae 1986; Nakae and Tachibana 1986), and the occurrence of several cavities in the MCs, ICs, and BCs (arrow 4) (Lee et al. 2020). In addition, the walls of VSV were also thickened (arrow 5) (Fukushima et al. 2005, 2006; Jorgensen 1961). The damage to the inner ear nervous system caused by hyperglycemia is mainly atrophy (arrow 6) (Makishima and Tanaka 1971) or loss (arrow 7) (Lee et al. 2008, 2020; Makishima and Tanaka 1971; Kang et al. 2020) of SGNs, loss of afferent nerve fibers (arrow 9), and swelling and dislocation of fiber endings (arrow 8) (Kang et al. 2020). The damage to the OC caused by hyperglycemia is mainly manifested as loss (arrow 10) (Fukushima et al. 2005, 2006; Nakae and Tachibana 1986; Triana et al. 1991; Rust et al. 1992) or degeneration (occasional occurrence of a vacuole) (arrow 11) (Nakae and Tachibana 1986) of OHCs (arrow 11) and the disappearance of the nucleus of SCs (arrow 12) (Lee et al. 2008).

We also present the pathological features of the inner ear affected by DM in human temporal bone studies and animal model studies in Tables 1 and 2, respectively.

## Protective factors against DRHL

Interventions for DRHL are still being vigorously explored. Several researchers have discussed possible treatments from the perspective of glycemic control and antioxidant therapy.

### Glycemic control and DRHL

It is unclear whether patients with DRHL can recover hearing after strict glycemic control. To date only a few studies have reported the effects of glycemic control on hearing recovery in SSNHL patients with DM.

Ryu et al. (2014, 2017). After intensive SSNHL treatment and strict glycemic control, the rate of hearing recovery in DM patients was very high. For example, hearing recovery rates in the non-diabetic group, the prediabetic group, and the DM group were 63.4%, 38.2%, and 80.0%, respectively. In contrast, Park et al. showed that the degree of glycemic control before and after the onset of SSNHL had no significant impact on the hearing recovery of DM patients. Among them, the hearing gain of the affected ear was 23.4 ± 18.7 dB and 25.6 ± 23.7 dB in the well-controlled group (mean blood glucose < 200 mg/dL) and the poorly-controlled group (mean blood glucose ≥ 200 mg/dL) (*P* = 0.494), respectively, while the hearing recovery rates of the two groups were 30.3% and 36.8% (*P* = 0.409), respectively (Park et al. 2021). The difference between the two studies may be due to the shorter follow-up period (4 weeks) and a more stringent definition of hearing recovery in the study by Park et al.

### Antioxidant and DRHL

#### Astaxanthin and DRHL

Astaxanthin (AST) is a non-provitamin A carotenoid with a strong antioxidant effect, and its ability to quench singlet oxygen and capture free radicals is more than 10 times greater than that of β-carotene. Toprak et al. showed that compared with non-diabetic rats, rats in the T1DM group had lower DPOAE and higher ABR (ABR: 35.8 ± 3.27 vs. 26.5 ± 1.41), decreased antioxidant enzyme levels and increased proinflammatory cytokine levels in cochlear tissue. Compared with T1DM rats, the hearing function of T1DM rats was recovered after AST treatment (DPOAE was increased and ABR was decreased (27.7 ± 2.91). In this treatment group, the antioxidant oxidase level in cochlear tissue was increased, and the proinflammatory cytokine level in cochlear tissue was significantly decreased (*P* < 0.05) (Toprak and Dedeoglu 2022).

#### Alpha-lipoic acid and DRHL

Alpha-lipoic acid (ALA) is called “a universal antioxidant” (Packer et al. 1995). It has four antioxidant properties: chelating metals, scavenging ROS, regenerating endogenous antioxidants, and repairing oxidative damage (Devasagayam et al. 1993; Kagan et al. 1992). Previous experiments have shown that ALA is safe and effective in the treatment of diabetic neuropathy (Papanas and Ziegler 2014) and has a protective effect against hearing loss caused by ototoxic drugs (Kim et al. 2018). In a zebrafish experiment, Kim et al. showed that the number of surviving hair cells in zebrafish larvae in the T1DM group was lower than that in the non-diabetic group. However, the survival number of hair cells of juvenile zebrafish treated with ALA was significantly higher than that of the corresponding no-ALA treatment group (*P* < 0.05) (Kim et al. 2021). This study suggests that ALA may be a potential agent to reduce cochlear hair cell damage in diabetic patients.

#### Asiaticoside and DRHL

Asiaticoside (AC) is a triterpene glycoside isolated from the medicinal plant *Centella asiatica* that stimulates collagen synthesis and is used for cosmetic treatments and repairing skin wounds (Liu et al. 2021) and has recently been found to have neuroprotective effects (Qi et al. 2014). In recent years, Xing et al. (2017).

These studies suggest that AC may reverse the harmful effects of DM on hearing. However, these studies have only been performed in animal models. More clinical studies are needed.

## Conclusion

The harmful effects of DM on hearing have been reported in many studies. DM will not only increase the prevalence of hearing loss, but also aggravate deafness. Therefore, urgent attention needs to be paid to such harmful effects. Based on previous studies, we further concluded that patients with DM have a worse hearing threshold than normal people, and the main affected frequency will gradually progress from high to low frequency with age. However, the effect of DM on hearing is most prominent between 35 and 65 years old. The duration and severity of DM act as important risk factors in the progression of DRHL. Comparatively, there is no strong evidence that different types of DM (T1DM and T2DM) have distinct effects on hearing loss. In addition, several new risk factors have emerged, such as taking PPI or vitamin D deficiency. However, treatments for DRHL are still being explored. The effectiveness of glycemic control for hearing recovery in diabetic patients remains controversial. It seems that intensive SSNHL treatment combined with strict glycemic control can improve the hearing recovery rate of diabetic patients with this particular type of SNHL. From the perspective of antioxidants, some researchers have observed that AST, ALA, and AC can protect hearing in diabetic animals, such as reducing the degeneration of hair cells, improving the level of antioxidant enzymes in cochlear tissue, and restoring hearing function. If these treatments prove to be effective in diabetic patients, they are expected to become an adjunctive therapeutic option for patients with DRHL.

## Data Availability

Not applicable.

## Abbreviations

ABR: Auditory brainstem-evoked response
AC: Asiaticoside
ALA: Alpha-lipoic acid
AST: Astaxanthin
BCs: Basal cells
CAT: Catalase
DM: Diabetes mellitus
DRHL: Diabetes-related hearing loss
FBG: Fasting blood glucose
GPx: Glutathione peroxidase
HbA1c: Glycosylated hemoglobin
ICs: Intermediate cells
IHCs: Inner hair cells
MCs: Marginal cells
OAE: Otoacoustic emissions
OC: Organ of Corti
OHCs: Outer hair cells
PTA: Pure tone audiometry
ROS: Reactive oxygen species
SCs: Supporting cells
SGNs: Spiral ganglion neurons
SL: Spiral ligament
SMA: Spiral modiolar artery
SNHL: Sensorineural hearing loss
SOD: Superoxide dismutase
SSNHL: Sudden sensorineural hearing loss
SV: Stria vascularis
T1DM: Type 1 diabetes mellitus
T2DM: Type 2 diabetes mellitus
VSV: Vessels of the stria vascularis

## References

[CR6] Akinpelu OV, Mujica-Mota M, Daniel SJ (2014). Is type 2 diabetes mellitus associated with alterations in hearing? A systematic review and meta-analysis. Laryngoscope.

[CR46] Akyay A, Soylu E, Unsal S, Demirol H, Bahceci S (2021). Hearing status in vitamin B12-deficient children. J Paediatr Child Health.

[CR25] Al-Rubeaan K (2021). Hearing loss among patients with type 2 diabetes mellitus: a cross-sectional study. Ann Saudi Med.

[CR29] Alizadeh Y, Jalali MM, Sehati A (2022). Association of different severity of diabetic retinopathy and hearing loss in type 2 diabetes mellitus. Am J Otolaryngol.

[CR4] Axelsson A, Sigroth K, Vertes D (1978). Hearing in diabetics. Acta Otolaryngol Suppl.

[CR40] Bainbridge KE, Hoffman HJ, Cowie CC (2011). Risk factors for hearing impairment among U.S. adults with diabetes: National Health and Nutrition Examination Survey 1999–2004. Diabetes Care.

[CR51] Bener A, et al. The impact of vitamin D Deficiency on Retinopathy and hearing loss among type 2 Diabetic patients. Biomed Res Int. 2018;2018. 2714590. 10.1155/2018/2714590 PMC6077590 30112372

[CR60] Breckenridge A, Welborn TA, Dollery CT, Fraser R (1967). Glucose tolerance in hypertensive patients on long-term diuretic therapy. Lancet.

[CR28] Celik O, Yalcin S, Celebi H, Ozturk A (1996). Hearing loss in insulin-dependent diabetes mellitus. Auris Nasus Larynx.

[CR20] Chee J, Kuah D, Loh WS, Loo JHY, Goh X (2022). Diabetes is a risk factor for hearing loss in older adults: results of a community screening programme. Clin Otolaryngol.

[CR32] Cruickshanks KJ (2015). Smoking, central adiposity, and poor glycemic control increase risk of hearing impairment. J Am Geriatr Soc.

[CR45] Curhan SG (2015). Carotenoids, vitamin A, vitamin C, vitamin E, and folate and risk of self-reported hearing loss in women. Am J Clin Nutr.

[CR18] Das A (2018). Impairment of extra-high frequency auditory thresholds in subjects with elevated levels of fasting blood glucose. J Otol.

[CR79] Devasagayam TP, Subramanian M, Pradhan DS, Sies H (1993). Prevention of singlet oxygen-induced DNA damage by lipoate. Chem Biol Interact.

[CR3] Dillmann WH (2019). Diabetic Cardiomyopathy. Circ Res.

[CR19] Frisina ST, Mapes F, Kim S, Frisina DR, Frisina RD (2006). Characterization of hearing loss in aged type II diabetics. Hear Res.

[CR62] Fukushima H (2005). Cochlear changes in patients with type 1 diabetes mellitus. Otolaryngol Head Neck Surg.

[CR63] Fukushima H (2006). Effects of type 2 diabetes mellitus on cochlear structure in humans. Arch Otolaryngol Head Neck Surg.

[CR70] Gladney JH (1978). Experimental diabetes and the inner ear. A proposed biologic model. Ann Otol Rhinol Laryngol.

[CR24] Gupta S, Eavey RD, Wang M, Curhan SG, Curhan GC (2019). Type 2 diabetes and the risk of incident hearing loss. Diabetologia.

[CR42] Han WK, et al. Susceptibility of Diabetic mice to noise trauma. Biomed Res Int. 2018;2018. 7601232. 10.1155/2018/7601232 PMC5830016 29619376

[CR44] Hatano M, Uramoto N, Okabe Y, Furukawa M, Ito M (2008). Vitamin E and vitamin C in the treatment of idiopathic sudden sensorineural hearing loss. Acta Otolaryngol.

[CR12] Horikawa C (2013). Diabetes and risk of hearing impairment in adults: a meta-analysis. J Clin Endocrinol Metab.

[CR23] Hosseini MS, Saeedi M, SA KH (2020). Prevalence of hearing Disorders among type 2 diabetes Mellitus patients with and without vitamin D Deficiency. Maedica (Bucur).

[CR65] Jorgensen MB (1961). The inner ear in diabetes mellitus. Histological studies. Arch Otolaryngol.

[CR49] Jung CH (2016). Relationship between vitamin D status and vascular complications in patients with type 2 diabetes mellitus. Nutr Res.

[CR80] Kagan VE (1992). Dihydrolipoic acid–a universal antioxidant both in the membrane and in the aqueous phase. Reduction of peroxyl, ascorbyl and chromanoxyl radicals. Biochem Pharmacol.

[CR33] Kang SH (2016). Association between HbA1c Level and hearing impairment in a nondiabetic Adult Population. Metab Syndr Relat Disord.

[CR73] Kang KW, Pangeni R, Park J, Lee J, Yi E (2020). Selective loss of calretinin-poor cochlear afferent nerve fibers in Streptozotocin-Induced Hyperglycemic mice. J Nanosci Nanotechnol.

[CR2] Kanwar YS, Sun L, Xie P, Liu FY, Chen S (2011). A glimpse of various pathogenetic mechanisms of diabetic nephropathy. Annu Rev Pathol.

[CR61] Kariya S (2010). Comparing the cochlear spiral modiolar artery in type-1 and type-2 diabetes mellitus:a human temporal bone study. Acta Med Okayama.

[CR53] Kekilli M, Tanoglu A, Ocal S, Beyazit Y (2014). Rabeprazole-Induced Tinnitus. Ann Pharmacother.

[CR82] Kim KH (2018). Evaluating protective and therapeutic effects of alpha-lipoic acid on cisplatin-induced ototoxicity. Cell Death Dis.

[CR83] Kim E, Lee DW, Park HC, Kim DH (2021). Protective effects of alpha-lipoic acid on hair cell damage in diabetic zebrafish model. Mol Genet Metab Rep.

[CR1] Kollias AN, Ulbig MW (2010). Diabetic retinopathy: early diagnosis and effective treatment. Dtsch Arztebl Int.

[CR17] Kurien M, Thomas K, Bhanu TS (1989). Hearing threshold in patients with diabetes mellitus. J Laryngol Otol.

[CR71] Lee HS, Kim KR, Chung WH, Cho YS, Hong SH (2008). Early sensorineural hearing loss in ob/ob mouse, an animal model of type 2 diabetes. Clin Exp Otorhinolaryngol.

[CR7] Lee YY et al. (2020) Type 1 Diabetes Induces Hearing Loss: Functional and Histological Findings in An Akita Mouse Model. *Biomedicines* 8. 10.3390/biomedicines8090343 PMC7555388 32932780

[CR13] Lin SW, Lin YS, Weng SF, Chou CW (2012). Risk of developing sudden sensorineural hearing loss in diabetic patients: a population-based cohort study. Otol Neurotol.

[CR84] Liu L (2021). Asiaticoside-laden silk nanofiber hydrogels to regulate inflammation and angiogenesis for scarless skin regeneration. Biomater Sci.

[CR56] Lloyd-Mostyn RH, Lord IJ (1971). Ototoxicity of intravenous frusemide. Lancet.

[CR50] Luo BA, Gao F, Qin LL. (2017) The Association between Vitamin D Deficiency and Diabetic Retinopathy in Type 2 Diabetes: A Meta-Analysis of Observational Studies. *Nutrients* 9. 10.3390/nu9030307 PMC5372970 28335514

[CR16] Ma F, Gomez-Marin O, Lee DJ, Balkany T (1998). Diabetes and hearing impairment in mexican american adults: a population-based study. J Laryngol Otol.

[CR64] Makishima K, Tanaka K (1971). Pathological changes of the inner ear and central auditory pathway in diabetics. Ann Otol Rhinol Laryngol.

[CR52] Makunts T, Alpatty S, Lee KC, Atayee RS, Abagyan R (2019). Proton-pump inhibitor use is associated with a broad spectrum of neurological adverse events including impaired hearing, vision, and memory. Sci Rep.

[CR41] McQueen CT (1999). Non-insulin-dependent diabetic microangiopathy in the inner ear. J Laryngol Otol.

[CR27] Mishra A, Poorey VK (2019). Clinical and Audiometric Assessment of hearing loss in diabetes Mellitus. Indian J Otolaryngol Head Neck Surg.

[CR14] Mitchell P (2009). Relationship of type 2 diabetes to the prevalence, incidence and progression of age-related hearing loss. Diabet Med.

[CR10] Mozaffari M, Tajik A, Ariaei N, Ali-Ehyaii F, Behnam H (2010). Diabetes mellitus and sensorineural hearing loss among non-elderly people. East Mediterr Health J.

[CR11] Mujica-Mota MA, Patel N, Saliba I (2018). Hearing loss in type 1 diabetes: are we facing another microvascular disease? A meta-analysis. Int J Pediatr Otorhinolaryngol.

[CR67] Nakae S, Tachibana M (1986). The cochlea of the spontaneously diabetic mouse. II. Electron microscopic observations of non-obese diabetic mice. Arch Otorhinolaryngol.

[CR48] Norman AW, Frankel JB, Heldt AM, Grodsky GM (1980). Vitamin D deficiency inhibits pancreatic secretion of insulin. Science.

[CR9] Oh IH (2014). Hearing loss as a function of aging and diabetes mellitus: a cross sectional study. PLoS ONE.

[CR22] Okhovat SA (2011). Evaluation of hearing loss in juvenile insulin dependent patients with diabetes mellitus. J Res Med Sci.

[CR30] Ooley C (2017). Correlational Study of Diabetic Retinopathy and hearing loss. Optom Vis Sci.

[CR78] Packer L, Witt EH, Tritschler HJ (1995). Alpha-lipoic acid as a biological antioxidant. Free Radic Biol Med.

[CR81] Papanas N, Ziegler D (2014). Efficacy of alpha-lipoic acid in diabetic neuropathy. Expert Opin Pharmacother.

[CR76] Park E, Shim J, Choi SJ, Jung HH, Im GJ (2021). Effects of Glycemic Control on hearing outcomes in diabetes patients with idiopathic sudden Sensorineural hearing loss. J Int Adv Otol.

[CR85] Qi FY (2014). Neuroprotective effects of Asiaticoside. Neural Regen Res.

[CR58] Quick CA, Hoppe W (1975). Permanent deafness associated with furosemide administration. Ann Otol Rhinol Laryngol.

[CR43] Raynor EM, Carrasco VN, Prazma J, Pillsbury HC (1995). An assessment of cochlear hair-cell loss in insulin-dependent diabetes mellitus diabetic and noise-exposed rats. Arch Otolaryngol Head Neck Surg.

[CR39] Ren H (2017). Hearing loss in type 2 diabetes in Association with Diabetic Neuropathy. Arch Med Res.

[CR69] Rust KR, Prazma J, Triana RJ, Michaelis OEt, Pillsbury HC (1992). Inner ear damage secondary to diabetes mellitus. II. Changes in aging SHR/N-cp rats. Arch Otolaryngol Head Neck Surg.

[CR74] Ryu OH (2014). Hyperglycemia as a potential prognostic factor of idiopathic sudden sensorineural hearing loss. Otolaryngol Head Neck Surg.

[CR75] Ryu OH (2017). Insulin effect on hearing recovery in idiopathic sudden sensorineural hearing loss: retrospective study of 145 patients. Clin Otolaryngol.

[CR31] Schade DS (2018). Hearing impairment and type 1 diabetes in the Diabetes Control and Complications Trial/Epidemiology of Diabetes Interventions and Complications (DCCT/EDIC) Cohort. Diabetes Care.

[CR47] Schmitz J (2012). Vitamin a supplementation in preschool children and risk of hearing loss as adolescents and young adults in rural Nepal: randomised trial cohort follow-up study. BMJ.

[CR55] Schwartz GH (1970). Ototoxicity of furosemide. N Engl J Med.

[CR8] Seo YJ (2022). Circulatory disturbance of the cochlear spiral modiolar artery in a type 2 diabetic mouse model. Laryngoscope Investig Otolaryngol.

[CR35] Shafiepour M, Bamdad Z, Radman M (2022). Prevalence of hearing loss among patients with type 2 diabetes. J Med Life.

[CR72] Sharma K, Seo YW, Yi E (2018). Differential expression of ca(2+)-buffering protein calretinin in Cochlear Afferent fibers: a possible link to vulnerability to traumatic noise. Exp Neurobiol.

[CR15] Shen Y (2021). Association of Glycosylated Hemoglobin A1c Level with Sudden Sensorineural hearing loss: a prospective study. Front Endocrinol (Lausanne).

[CR21] Sieger A, White NH, Skinner MW, Spector GJ (1983). Auditory function in children with diabetes mellitus. Ann Otol Rhinol Laryngol.

[CR26] Srinivas CV, Shyamala V, Shiva Kumar BR (2016). Clinical study to evaluate the Association between Sensorineural hearing loss and diabetes Mellitus in Poorly Controlled Patients whose HbA1c > 8. Indian J Otolaryngol Head Neck Surg.

[CR66] Tachibana M, Nakae S (1986). The cochlea of the spontaneously diabetic mouse. I. Electron microscopic observation of KK mice. Arch Otorhinolaryngol.

[CR37] Taylor IG, Irwin J (1978). Some audiological aspects of diabetes mellitus. J Laryngol Otol.

[CR5] Teng ZP (2017). An association of type 1 diabetes mellitus with auditory dysfunction: a systematic review and meta-analysis. Laryngoscope.

[CR77] Toprak SF, Dedeoglu S (2022). Astaxanthin protects against hearing impairment in diabetic rats. Braz J Otorhinolaryngol.

[CR68] Triana RJ (1991). Inner ear damage secondary to diabetes mellitus. I. Changes in adolescent SHR/N-cp rats. Arch Otolaryngol Head Neck Surg.

[CR36] Uchida Y, Sugiura S, Ando F, Nakashima T, Shimokata H (2010). Diabetes reduces auditory sensitivity in middle-aged listeners more than in elderly listeners: a population- based study of age-related hearing loss. Med Sci Monit.

[CR57] Venkateswaran PS (1971). Transient deafness from high doses of frusemide. Br Med J.

[CR38] Wang W (2022). Sex-specific Associations between Diabetes Mellitus and hearing loss in the Middle-Aged and Elderly individuals: a National Cohort study of chinese adults. Endocr Pract.

[CR59] Weller JM, Borondy M (1967). Effect of furosemide on glucose metabolism. Metabolism.

[CR86] Xing Y (2017). Asiaticoside protects cochlear hair cells from high glucose-induced oxidative stress via suppressing AGEs/RAGE/NF-kappaB pathway. Biomed Pharmacother.

[CR54] Yee J, Han HW, Gwak HS (2022). Proton pump inhibitor use and hearing loss in patients with type 2 diabetes: evidence from a hospital-based case-control study and a population-based cohort study. Br J Clin Pharmacol.

[CR34] Zeng GH, Su YJ, Liu HY, Lu DH (2022). Is sudden deafness in non-diabetic patients affected by their glycosylated hemoglobin levels?. Eur Rev Med Pharmacol Sci.

